# Prostate cancer risk prediction based on complete prostate cancer family history

**DOI:** 10.1002/pros.22925

**Published:** 2014-11-18

**Authors:** Frederick Albright, Robert A Stephenson, Neeraj Agarwal, Craig C Teerlink, William T Lowrance, James M Farnham, Lisa A Cannon Albright

**Affiliations:** 1 Department of Pharmacotherapy, Pharmacotherapy Outcomes Research Center, College of Pharmacy, University of UtahSalt Lake City, Utah; 2 Department of Surgery, Division of Urology, School of Medicine, University of UtahSalt Lake City, Utah; 3 George E. Wahlen Department of Veterans Affairs Medical CenterSalt Lake City, Utah; 4 Huntsman Cancer Institute, University of UtahSalt Lake City, Utah; 5 Department of Medicine, Division of Medical Oncology, University of UtahSalt Lake City, Utah; 6 Department of Internal Medicine, Division of Genetic Epidemiology, University of Utah School of MedicineSalt Lake City, Utah

**Keywords:** prostate cancer, familiality of cancer, genealogy, UPDB, cancer

## Abstract

**Background:**

Prostate cancer (PC) relative risks (RRs) are typically estimated based on status of close relatives or presence of any affected relatives. This study provides RR estimates using extensive and specific PC family history.

**Methods:**

A retrospective population-based study was undertaken to estimate RRs for PC based on complete family history of PC. A total of 635,443 males, all with ancestral genealogy data, were analyzed. RRs for PC were determined based upon PC rates estimated from males with no PC family history (without PC in first, second, or third degree relatives). RRs were determined for a variety of constellations, for example, number of first through third degree relatives; named (grandfather, father, uncle, cousins, brothers); maternal, paternal relationships, and age of onset.

**Results:**

In the 635,443 males analyzed, 18,105 had PC. First-degree RRs ranged from 2.46 (=1 first-degree relative affected, CI = 2.39–2.53) to 7.65 (=4 first-degree relatives affected, CI = 6.28–9.23). Second-degree RRs for probands with 0 affected first-degree relatives ranged from 1.51 (≥1 second-degree relative affected, CI = 1.47–1.56) to 3.09 (≥5 second-degree relatives affected, CI = 2.32–4.03). Third-degree RRs with 0 affected first- and 0 affected second-degree relatives ranged from 1.15 (≥1 affected third-degree relative, CI = 1.12–1.19) to 1.50 (≥5 affected third-degree relatives, CI = 1.35–1.66). RRs based on age at diagnosis were higher for earlier age at diagnoses; for example, RR = 5.54 for ≥1 first-degree relative diagnosed before age 50 years (CI = 1.12–1.19) and RR = 1.78 for >1 second-degree relative diagnosed before age 50 years, CI = 1.33, 2.33. RRs for equivalent maternal versus paternal family history were not significantly different.

**Conclusions:**

A more complete PC family history using close and distant relatives and age at diagnosis results in a wider range of estimates of individual RR that are potentially more accurate than RRs estimated from summary family history. The presence of PC in second- and even third-degree relatives contributes significantly to risk. Maternal family history is just as significant as paternal family history. PC RRs based on a proband's complete constellation of affected relatives will allow patients and care providers to make more informed screening, monitoring, and treatment decisions. *Prostate 75:390–398, 2015*. © 2014 Wiley Periodicals, Inc.

## Introduction

The American Cancer Society estimates that 233,000 men will be diagnosed with PC and 29,480 will die from PC in 2014 [1,2]. Current National Cancer Institute (NCI) Surveillance Epidemiology and End Results (SEER) data suggest a 15.3% lifetime risk for men born today in the US (based on data from 2008–2010) [2]. The latest 2011 SEER analysis gives a prevalence of 2,707,821 men alive with PC (in 2011) and 98.9% surviving at least 5 years. Age adjusted incidence is 147.8 new cases per 100,000 men per year [2]. Screening for PC has been associated with substantial over-diagnosis and over-treatment, but has been shown to reduce PC mortality [3].

PC family history is a substantial risk factor for PC. Estimated risks typically use only first-degree relatives or a less specific family history of close relatives [4,5], although some studies have presented familial risks based on PC in extended families including a familial risk assessment model [6,7]. Herein, estimated risk for PC was based on the constellation of a male proband's affected first-, second-, and third-degree relatives, the number of affected individuals, age at diagnosis of affected relatives, and paternal and maternal contribution. This analysis expands and refines published PC risk estimates based on family history [6–9] and provides individualized risk estimates based on more complete knowledge of PC family history.

## Materials and Methods

### Utah Population Database and Utah Cancer Registry

This study utilized a large population-based genealogy and phenotype resource (Utah Population Data Base or UPDB) [10]. The UPDB contains genealogical and population vital statistics records. The UPDB includes over 6.5 million individuals [11], 1,238,061 of whom who were analyzed in this study have genealogy data for both parents, all four grandparents, and at least six of eight great grandparents. The UPDB data is updated each year, and is current to 2012 for this analysis.

The UPDB has been linked to the Utah Cancer Registry (UCR) and other statewide medical and demographic databases [10–12]. The UCR was established in 1966; in 1973 it became an NCI SEER registry [13]. All independent primary cancers diagnosed or treated in Utah are reported to the UCR; all reported PCs have histologic confirmation [14]. The University of Utah Institutional Review Board approved this study.

Probands were defined as all males, regardless of their prostate cancer status, with a specific constellation of PC family history including: first-degree relatives (FDR), second- degree relatives (SDR), and third-degree relatives (TDR). These relationships are described in TableI.

**Table I:** Example Relationships by Degree of Relatedness (not all-inclusive)

Degree of relatives	Ancestors	Descendants	Other
First-degree (FDR)	Parents	Children	Siblings
Second-degree (SDR)	Grandparents	Grandchildren	Avunculars
Third-degree (TDR)	Great grandparents	Great grand children	1st cousins, great avunculars

### Estimation of Constellation Relative Risk (RR)

To estimate the RR for PC for a specific family history constellation, PC rates must be determined for the population. All males were assigned to 5-year birth year and birth state (Utah or not Utah) cohorts. Cohort-specific PC rates were calculated from the set of 201,791 males in the UPDB with no family history of PC (no first-, no second-, and no third-degree relatives affected with PC). Cohort specific rates (*r_i_*) were determined by counting the number of observed prostate cancer cases in each cohort (*c_i_*) divided by the total number of males in the cohort (*n*_i_) that is, *r_i_*=*c_i_*/ *n_i_*.

For each specific family history constellation of PC considered, all males who had the constellation were considered probands; the observed number of probands with PC was counted (observed cases, or *O*). The expected number of prostate cases (*E*) among the probands was calculated by applying the cohort-specific PC rates (estimated in the 201,791 males with no PC family history) to all of the probaids, and summing over all cohorts, as follows: 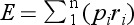, where *p_i_* refers to the number of probands in the constellation of cohort *i*, and *r_i_* is the cohort specific PC rate as described above. The estimated RR for PC for males with the specific family history constellation is the ratio of the number of observed cases (*O*) to the number of expected cases (*E*) among the probands that is, 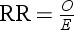. Assuming that the number of observed cases follows a Poisson distribution with mean equal to *E*, two-tailed 95% confidence intervals were constructed [15].

## Results

Among the 635,443 males with ancestral data present in the UPDB regardless of age, 18,105 (2.85%) had a diagnosis of PC in the Utah Cancer Registry. TableII shows the estimated rate of PC in all UPDB males (2.85%), the rate of PC in males with family history (3.50%), and in males without (1.45%) family history, where family history is defined as at least one affected first, second, or third-degree relative.

**Table II:** Rate of PC in All Family-History-Positive* and Family-History-Negative Males in the UPDB, Regardless of Age

Population description	Number in population	Percent of population	Nr. PC cases	% PC cases
All males	635,443	100	18,105	2.85
Males with family history of PC	433,652	68	15,180	3.50
Males without family history of PC	201,791	32	2,925	1.45

^*^Family history defined as 1 or more affected FDR, SDR, or TDR.

### Estimated RRs for First-Degree Relative (FDR) Constellations

TableIIIA shows estimated RRs for FDR family history constellations; SDR and TDR family history are ignored. TableIIIA shows the number of probands with the specific family history (n), the number of probands with PC (Obs), the expected number of probands with PC (Exp), the RR estimate, the significance (*P*-value) and the two-tailed 95% confidence lower (L) and upper (U) bounds. The estimated RR for PC in males with 0 FDRs affected is 1.20 (CI 1.18, 1.23). This reflects the increased risk due to the presence of affected SDRs and TDRs for probands with no FDR family history. While the RR for at least one affected FDR = 2.76 (2.69, 2.82), the more specific RRs based on the number of affected FDRs range from 2.46 (2.39, 2.53) for exactly one affected FDR to RR = 7.65 (6.28, 9.23) for exactly four affected FDRs.

**Table III:** Estimated RRs for PC Based on Proband's Family History for Degree of Relatives Affected Constellations (A–D), Age at Diagnosis (E,F), Maternal Versus Paternal Relative Risks (G), and Combined Maternal and Paternal Line PC Familial Constellations (H)

No. degree relatives affected	n	Obs	Exp	RR	*P*-value	L	U
A. Estimated RRs for prostate cancer based on proband's number of FDRs diagnosed with PC; SDR and TDR family history ignored							
=0	561,636	11,665	9,681.80	1.20	≤0.0001	1.18	1.23
=1	63,150	4,714	1,915.58	2.46	≤0.0001	2.39	2.53
≥1	73,807	6,439	2,336.64	2.76	≤0.0001	2.69	2.82
=2	8,718	1,272	342.47	3.71	≤0.0001	3.51	3.92
≥2	10,657	1,725	421.06	4.10	≤0.0001	3.91	4.29
=3	1,504	322	60.44	5.33	≤0.0001	4.76	5.94
≥3	1,939	453	78.60	5.76	≤0.0001	5.24	6.32
=4	333	109	14.25	7.65	≤0.0001	6.28	9.23
≥4	435	131	18.16	7.21	≤0.0001	6.03	8.56
=5	85	18	3.47	5.19	≤0.0001	3.08	8.21
≥5	102	22	3.91	5.63	≤0.0001	3.53	8.52
B. Estimated RRs for PC based upon the proband's number of SDRs diagnosed with PC; 0 FDR; TDR family history ignored							
≥1	149,885	3,981	2,629.26	1.51	≤0.0001	1.47	1.56
≥2	38,038	1,264	683.65	1.85	≤0.0001	1.75	1.95
≥3	11,204	411	186.15	2.21	≤0.0001	2.00	2.43
≥4	3,749	133	53.22	2.50	≤0.0001	2.09	2.96
≥5	1,323	54	17.48	3.09	≤0.0001	2.32	4.03
C. Estimated RRs for PC based upon the proband's number of TDRs diagnosed with PC; 0 FDRs; 0 SDRs							
=0	201,791	2,925	2,925.00	1.00	1.0000	0.96	1.04
≥1	209,960	4,759	4,127.54	1.15	≤0.0001	1.12	1.19
≥2	91,841	2,744	2,179.79	1.26	≤0.0001	1.21	1.31
≥3	39,435	1,451	1,100.89	1.32	≤0.0001	1.25	1.39
≥4	17,583	770	544.11	1.42	≤0.0001	1.32	1.52
≥5	8,246	386	257.52	1.50	≤0.0001	1.35	1.66
D. Estimated RRs for PC based upon the proband's number of affected SDRs; exactly 1 affected FDR; TDR family history ignored							
=0	35,437	2,626	1,169.29	2.25	≤0.0001	2.16	2.33
≥1	27,713	2,088	746.28	2.80	≤0.0001	2.68	2.92
≥2	13,524	1,391	355.20	3.92	≤0.0001	3.71	4.13
≥3	3,954	314	87.61	3.58	≤0.0001	3.20	4.00
≥4	1,532	122	27.99	4.36	≤0.0001	3.62	5.20
≥5	574	57	9.57	5.96	≤0.0001	4.51	7.72
E. Estimated RRs for PC for by youngest age of diagnosis for at least 1 affected FDR; SDR and TDR family history ignored							
<50	777	95	17.15	5.54	<0.0001	4.48	6.77
50–59	7,770	858	205.46	4.18	<0.0001	3.90	4.47
60–69	24,149	2,275	759.51	3.00	<0.0001	2.87	3.12
70–79	28,918	2,361	952.25	2.48	<0.0001	2.38	2.58
79+	12,193	850	402.28	2.11	<0.0001	1.97	2.26
F. Estimated RRs for PC for at least 1 affected SDR by youngest age at diagnosis for 0 FDRs; TDR family history ignored							
<50	1,571	53	29.80	1.78	<0.0001	1.33	2.33
50–59	16,844	453	284.97	1.59	<0.0001	1.45	1.74
60–69	53,049	1,336	828.02	1.61	<0.0001	1.53	1.70
70–79	56,177	1,462	968.77	1.51	<0.0001	1.43	1.59
79+	22,244	677	517.71	1.31	<0.0001	1.21	1.41
G. Estimated RRs for maternal versus paternal PC family history constellations							
Mother's father affected	25,991	132	61.19	2.16	<0.0001	1.80	2.56
Father's father affected	24,917	89	46.09	1.93	<0.0001	1.55	2.38
Mother's brother affected	44,435	2,228	1,205.26	1.85	<0.0001	1.77	1.93
Father's brother affected	44,378	2,036	1,073.61	1.90	<0.0001	1.81	1.98
Sister's son affected	34,187	1,942	869.41	2.23	<0.0001	2.14	2.34
Brother's son affected	32,238	1,809	756.70	2.39	<0.0001	2.28	2.50
H. Estimates of RR for PC for combined maternal and paternal family history constellations							
Mother's father and father's father affected	2,366	9	1.73	5.20	<0.0001	2.38	9.87
At least 1 affected mother's brother and at least 1 affected father's brother	6,322	378	158.45	2.39	<0.0001	2.15	2.64

### Estimated RRs for Second-Degree Relative (SDR) Constellations

TableIIIB shows estimated RRs for PC based on a proband's SDR family history, with 0 affected FDRs and ignoring TDR family history. In the absence of affected FDRs, the presence of any number of affected SDRs is associated with significantly increased risk for PC: RR = 1.51(1.47, 1.56) with ≥1 affected SDR to RR = 3.09 (2.32, 4.03) for ≥5 affected SDRs.

### Estimated RRs for Third-Degree Constellations

TableIIIC shows estimated RRs for PC based on TDR family history for probands with 0 FDRs and 0 SDRs. Because the baseline risk for PC was estimated from the 201,791 men with 0 FDRs, 0 SDRs, and 0 TDRs, the estimated RR for 0 TDRs (TableIIIC) = 1.00 (0.96, 1.04). Any number of affected TDRs is associated with significantly increased risk; with a RR for one affected TDR = 1.15 (1.12, 1.19); increasing to five or more affected TDRs RR = 1.50 (1.35, 1.66).

All possible constellations of PC family history are too numerous to include; only limited examples are presented. In order to demonstrate the contribution of SDR family history in the presence of FDR family history, TableIIID presents SDR PC constellations with exactly one FDR affected and TDR family history ignored. The RRs ranged from 2.25 (for 0 SDRs) to 5.96 (for ≥5 SDRs), demonstrating that number of affected SDRs contributes to accurate risk prediction even in the presence of a positive FDR family history. The RR for at least five SDRs in the presence of exactly one affected FDR (RR = 5.96) approaches twice the risk of at least five SDRs with no FDRs affected (RR = 3.09).

### Estimated RRs for Family History Constellations Including Youngest Diagnosis Age of Affected Relatives

TableIIIE shows estimated RRs for probands having at least one affected FDR, with SDRs and TDRs ignored, while considering the youngest age at diagnosis among the affected FDRs. For probands with an FDR whose age at diagnosis was less than 50 years, RR = 5.54 (4.48, 6.77); this is more than twice the risk for a proband with at least one affected FDR whose age at diagnosis is not considered (RR = 2.76; TableIIIA).

TableIIIF shows estimated RRs for at least one affected SDR by age at diagnosis of the youngest affected SDR, with 0 affected FDRs, and TDRs ignored. When the age at diagnosis for the youngest affected SDR is less than 50 years the RR = 1.78 compared to RR = 1.51 for the same family history constellation when age at diagnosis of the SDR relative is not considered (TableIIIB).

### Estimated RRs for PC Constellations Including Maternal and Paternal Family History

Often estimates of RR for PC do not consider maternal family history. Three different constellations of equivalent family history for maternal and paternal lines are shown in TableIIIG; all other family history is ignored. For all three constellations, there is no significant difference in the risk estimates based on maternal versus paternal contribution; all show significantly elevated risks. Estimated RRs are also shown in TableIIIH for two combined maternal and paternal constellations. Although the two RRs differ substantially (RR = 5.20 and 2.39), each of these two constellations is equivalent to FDR and TDR ignored and ≥2 SDR (RR = 2.56, CI = 2.47, 2.65; data not shown) and both CIs include this estimate.

**Figure 1:**
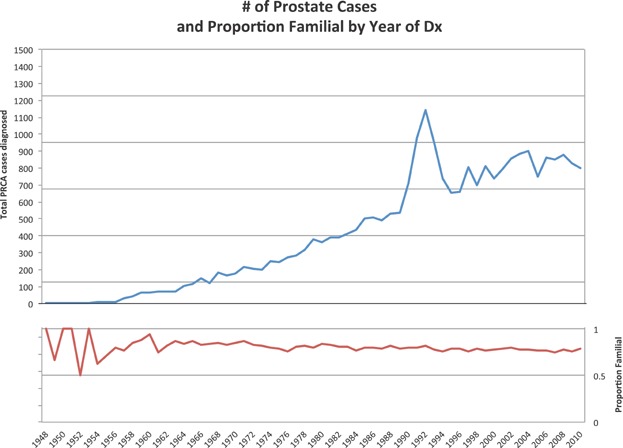
Number of PC diagnoses by year of diagnosis in UCR, and the percent of PC cases by year of diagnosis who are “familial” (with at least one affected FDR, SDR, or TDR).

It has been suggested that increased diagnostic activity, especially in the PSA era, can contribute to the increased risk of diagnosis of PC in relatives and lead to bias of estimated risks for relatives [16]. We have considered this possibility in the UPDB. Figure 1 shows the number of prostate cancer diagnoses by diagnosis year, as well as the percent of these cases that are termed “familial” (at least one affected FDR, SDR, or TDR) since the existence of the UCR. The increase in PC diagnoses in the PSA era is quite obvious, as is the observation that the percent of familial PC cases has not increased over this time period.

## Discussion

PC risk has been related to numerous factors, including family history, diet, environmental exposures, age, ethnicity, and obesity. Familial risk for PC is comprised of a combination of inherited genetic and shared environmental risk factors. Twin studies demonstrate that genetic risk accounts for approximately 42% of total familial risk for PC (higher than for any other cancer studied) [17].

Of the known PC risk factors, genetic risk is appealing for investigation, but the identification of PC predisposition genes segregating in relatives has proven to be elusive given the genetic complexity of the disorder. In the meantime, individualized PC risk estimates based on a male's specific family history may prove to be an inexpensive and efficient mechanism to identify males at highest risk. Although there has been considerable discussion on what the appropriate screening for such men should be, it is clear that screening is desired by some, and has been effective in reducing PC mortality.

We have previously estimated RRs for PC in the UPDB [15,18,19] using the more traditional method of selecting all PC cases as probands and estimating risks to their relatives of different degrees. As expected, RRs estimated in these publications are typically much lower than the constellation RRs reported here. The alternative RR estimation method presented here was designed because traditional RRs do not consider the complete family history and may not be particularly useful for a male with a specific family PC constellation. The estimated RRs presented here are the first step towards a more individualized risk estimate for any male based on his current knowledge of his family history. It should be noted that a male's family history for PC will most often increase as he ages, and consideration of his updated family history should update his risk estimate for PC.

If one uses the common definition of a positive family history for prostate cancer (one or more affected first-degree relative(s) and ignoring all other relationships), the estimated familial rate for prostate cancer is 11.6% (TableIIIA, FDR ≥1) using the Utah data. In this analysis of the Utah population, 68% of males were identified as having a positive family history for PC using what we consider a more appropriate definition for a positive family history (consisting of at least one FDR, SDR, or TDR affected with prostate cancer). We identified significantly increased risk for various PC family history constellations, with significantly elevated RRs ranging from RR = 1.15 to RR > 7.0 in the constellations considered. This range of risk for PC may be surprising to those familiar with other studies of familial PC risk, but it demonstrates the power of the UPDB resource for accurate estimation of risk for specific family history constellations.

Although a majority (68%) of the Utah male population has measurable and significantly elevated familial risk of prostate cancer, the risk is substantial in only a minority of males. According to our data, 26% of males in Utah have at least a two fold increased risk for PC based on family history alone, and 10% have at least a three fold risk increased risk (TableIV).

**Table IV:** Minimal Family History Constellations Associated With Estimated RR > 2.0 and >3.0 for PC

RR > 2.0 (26% of males)	RR > 3.0 (10% of males)
>0 affected FDR	>1 affected FDR
>2 affected SDRs	>4 affected SDRs
Mother's father affected	Both grandfathers affected
Nephew affected	>0 affected FDRs and >1 affected SDR
Maternal and paternal uncles affected	>0 FDR and dx <70 years

This analysis of a large population-based resource used a distinctive approach to estimate RRs for PC. Specifically the estimated risks are based on a comprehensive picture of prostate cancer family history for an individual and are therefore likely to be more accurate than those typically reported or estimated using less, or less specific, family history data. This approach is based on: (i) the inclusion of available family history data for close and more distant relatives, age at diagnosis, and maternal versus paternal contribution; (ii) uniform and statewide PC phenotype data; and (iii) absence of dependence on recall for relationships and for the PC phenotype. Further, all PC cases recorded in the UCR were histologically confirmed.

It has been demonstrated that family history in combination with genetic marker data for variants associated with increased PC risk contains more information about disease risk than does genetic marker data alone. MacInnis [20] showed that the combination of family history with genotypes from 25 known SNPs associated with PC risk in GWAS far outperformed prediction of prostate cancer using those SNPs alone [20]. According to the model from that study, a typical 50-year-old male PC risk without a family history of PC who carried 99% of the risk alleles from 25 associated SNPs had a 30% risk of developing PC by age 85, or almost twice population lifetime risk (17.8% in the US according to Raymond, et al. [21]). However, for a typical 50 year old male with two affected first degree relatives who carried 99% of the risk alleles from 25 associated SNPs, the probability of developing PC by age 85 was 100%. Until the clinical validity of risk markers derived via GWAS for disease prediction has been demonstrated, as some have warned is necessary [22], family history data remains an economically sustainable, viable, powerful, and effective alternative for accurate PC risk estimation. Risk estimates should arguably be made using the most complete PC family history data available.

As expected, first-degree family history contributes most significantly to PC risk (TableIIIA), but other more distant family history effects were also observed (TablesIIIB–D). A second-degree relative family history, even in the absence of affected FDRs significantly elevates risk. Males with three or more affected SDR relatives, even in the absence of affected FDRs (RR = 2.21) are at similar risk to males with exactly one affected FDR (RR = 2.46). Earlier age at diagnosis of an affected relative increases risk significantly as age at diagnosis decreases, for both FDR and SDR affected relatives (TableIIIE, F). RRs appear to be equivalent whether the family history is maternal or paternal, suggesting that the maternal family history should never be ignored for PC (TableIIIG).

The estimated RR for a combined maternal and paternal family history, for example both mother's father and father's father affected (TableIIIH, RR = 5.20), is higher than the sum of the RRs for each category separately (RR = 2.16 and RR = 1.93, respectively; TableIIIG), suggesting a synergistic effect on risk. Such an effect was not observed when the combined family history consisted of at least one mother's brother (RR = 1.85) and at least one father's brother (RR = 1.90) for which the combined RR estimate = 2.39. This effect should be investigated further.

TableIV provides a summary of those family history constellations with RR ≥ 2, and separately for those constellations with RR ≥ 3, as a quick clinical reference for probands whose family history matches one of the constellations that fits the highest risk categories identified. These risk estimates are presented as data to which both clinician and patient can refer, in order to assist in determination of an appropriate PC screening plan.

Other risk data known about the patient could be combined with the RR estimates presented here in any decision making process. This might be especially important in the event SNP data are available for the patient [23,24]. Future work in this unique population resource includes model development to include even more specific family history variables and inclusion of other risk factors in the proband, for example, SNP variants, body mass index; as well as clinical characteristics of the PC in affected relatives (e.g., Gleason score or death due to prostate cancer).

There are limitations to this study. The UPDB genealogy data represents Utah pioneer founders and their descendants. Any PC cancers diagnosed out of state are censored, as are those occurring before 1966. Failure to link existing genealogy or cancer data could also result in data censoring. Such data censoring is more likely to lead to conservative, than exaggerated, risk estimates.

Currently the clinical relevance of these findings is limited by the fact that there is no consensus on prostate cancer screening. The results of the PLCO and ERSPC randomized prostate cancer screening trials have led to concerns about the utility of PSA screening and have questioned whether the harms of widespread screening surpass the benefits of early detection. The USPSTF recommends against screening, while the NCCN and AUA still acknowledge the potential benefit of PSA screening especially in patients at higher risk of prostate cancer. Both organizations recommend a man and his physicians discuss the risks and benefits of PSA screening and then make an informed decision about whether or not to pursue screening The knowledge of specific prostate cancer risk estimates associated with a man's detailed family history may further contribute to the selection of men who stand to benefit from targeted prostate cancer screening.

## Conclusions

The RR estimates presented are based on confirmed PC cancer data from the Utah population. The Utah founding population was primarily from Great Britain and Scandinavia, and has been shown to be genetically similar to the US and Northern Europe [25,26]. Inbreeding rates for Utah are similar to those estimated for the USA population (∽1.5%) or lower [27]. These RRs should be appropriate for Caucasian populations and males of Northern European origin and should not be extrapolated to other populations without validation.

In conclusion, these population-based estimates of PC risk are based on a male's specific constellation of PC family history compared to Utah population rates estimated for men without PC family history. These study results can be used to provide informative RRs for PC that are more precise for an individual than typical population summary risks which do not take complete PC family history into account. The results strongly imply the value of more in-depth family history for the patient and allow more individualized screening and awareness (e.g., educational preparedness). At the population level the methodology and results convey a potential to serve in modeling of the disease in the context of public health. This could have benefit in developing more applicable PC screening policies that can best identify and target those at highest risk of the disease. Using informative and more detailed family history in the planning of screening, treatment, and monitoring opens additional avenues for implementation of sound translational medicine practices, improving the quality of life in patients.
